# A Perspective on the 2023 Cholera Outbreaks in Zimbabwe: Implications, Response Strategies, and Policy Recommendations

**DOI:** 10.1007/s44197-023-00165-6

**Published:** 2023-11-27

**Authors:** Gbolahan Olatunji, Emmanuel Kokori, Abdulrahmon Moradeyo, Doyin Olatunji, Folake Ajibola, Oluwaseun Otolorin, Nicholas Aderinto

**Affiliations:** 1 Department of Medicine and Surgery, University of Ilorin, Ilorin, Nigeria; 2 Department of Medicine, Ladoke Akintola University of Technology, Ogbomoso, PMB 5000 Nigeria; 3 Department of Health Sciences, Western Illinois University, Illinois, USA; 4 Department of Medicine and Surgery, Ekiti State University, Ekiti, Nigeria

**Keywords:** Cholera, Zimbabwe, Public health, Africa

## Abstract

Cholera continues to severely threaten public health, particularly in regions with inadequate access to clean water and sanitation. Zimbabwe, a southern African nation, has witnessed recurrent cholera outbreaks, highlighting the enduring vulnerabilities faced by communities grappling with these underlying challenges. The 2023 cholera outbreak in Chegutu resulted in a devastating impact, with approximately 100 reported deaths and nearly 5000 confirmed and suspected cases. Beyond its immediate health consequences, the outbreak has strained the already fragile healthcare system, exacerbated issues of malnutrition, and disrupted education, particularly affecting vulnerable populations. The Zimbabwean government, non-governmental organisations (NGOs), and international agencies have initiated comprehensive efforts to combat the outbreak, encompassing medical treatment, surveillance, public health measures, infrastructure improvement, and community empowerment. Policy recommendations and future directions are discussed, emphasising risk communication, stakeholder engagement, standardisation, evaluation, resource allocation, and capacity-building to bolster prevention and control measures.

## Introduction

Cholera remains a persistent and severe threat to global public health, especially in regions lacking clean water and adequate sanitation facilities [1]. Zimbabwe, a southern African nation, has grappled with a recurring series of cholera outbreaks, underscoring the ongoing vulnerability of communities facing these underlying challenges [2].

In 2023, a cholera outbreak emerged in Chegutu, located in the Mashonaland West Province, starting in February [3]. The Ministry of Health and Child Care (MoHCC) confirmed the first suspected case on February 15, followed by a second case on February 17 [4]. By February 26, Chegutu district reported 37 suspected cholera cases, including 2 confirmed positive cases and no recorded deaths. However, by April 18, 2023, the situation had deteriorated significantly, with 579 suspected cholera cases, including 102 culture-confirmed cases and nine documented deaths. Alarming proportions were reached by August 27, 2023, resulting in 3894 cholera cases and 96 fatalities. Notably, this pandemic extended its reach to all ten provinces across the country, with Harare (1616), Mutare (1534), and Bulawayo (324) reporting the highest cumulative cases, accounting for 89% of the total cases.

In response to the escalating crisis, the Zimbabwean government implemented several measures to contain the disease's spread. These included restricting funeral attendance to 50 people, banning food sharing, and suspending social gatherings in vulnerable areas [5]. These measures were informed by past outbreaks, notably the largest in 2008–2009, which resulted in 98,592 illnesses and 4288 deaths [6]. Zimbabwe's recurring challenges, including water scarcity and deteriorating sanitation infrastructure, have paved the way for recent cholera outbreaks. Certain regions, such as Harare and Bulawayo, have endured months without running water, with raw sewage becoming a common sight in townships [6]. These factors, compounded by malnutrition, underdeveloped healthcare systems, and insufficient vaccination coverage, significantly contribute to the continued spread of cholera in the country.

The significance of examining the 2023 cholera outbreak in Zimbabwe goes beyond its immediate impact on the nation's health system. It also relates to the broader context of cholera epidemiology and the global fight against preventable waterborne diseases. Zimbabwe's recurring battle with cholera has served as a litmus test for assessing the resilience of health infrastructure, the effectiveness of emergency response, resource allocation, and public health management. This perspective aims to analyse the health implications of the 2023 Cholera Outbreak in Zimbabwe.

## Implications of Cholera in Affected Communities

The 2023 cholera outbreak in Zimbabwe has unfurled a profound and multifaceted impact on the communities it has struck, reaching far beyond the stark statistics of approximately 100 reported deaths and nearly 5000 confirmed and suspected cases [2].

### Health Impact

The profound health impact is the most immediate and pressing concern. The tragic death toll represents only a fraction of the suffering endured by those affected. Concurrent outbreaks of endemic diseases, such as malaria, stretch the already fragile healthcare systems to their limits. Malnutrition, particularly among children, is exacerbated due to the crisis. The scarcity of clean water amplifies the devastation, with cholera's dehydrating effects setting a vicious cycle of disease. Children, especially those from low-income families, are among the most vulnerable. They confront a dual challenge: limited access to healthcare and delayed arrival at healthcare facilities. Healthcare centres already operating with restricted resources and staffing are overwhelmed by the sudden surge of patients due to the cholera outbreak [7].

### Societal and Economic Consequences

The impact on society and the economy is multi-faceted. The bans on social gatherings in various regions have culminated in constraints on communal activities. In the Buhera district, strict limits on funeral attendance have been imposed, allowing no more than 50 attendees, and communal food sharing is prohibited [5]. In Harare, purchasing food from unlicensed vendors is no longer allowed, and adherence to social distancing measures, including refraining from physical contact like handshakes, is advised [5]. Schools, pivotal for children's education, have been severely affected, leading to disruptions in the learning process. Moreover, public advisories in regions such as Manicaland and Masvingo discourage attendance at open markets, social gatherings, and outdoor church camps, citing inadequate sanitation facilities. The psychological impact of these restrictions is significant, particularly in a world still grappling with the aftermath of the COVID-19 pandemic. The economic implications are substantial, especially in regions where large gatherings and market activities have been prohibited. The local economy in areas like Manicaland and Masvingo has endured a severe blow.

### Government Resource Reallocation

The financial burden of managing cholera outbreaks is substantial, especially in a country where the budget allocation falls 11% below the 15% benchmark established by the Abuja Declaration 2001 [8]. Critical resources have been redirected from other essential government projects to combat the outbreak. These resources have been sourced from various outlets, including the Government of Zimbabwe, the Reserve Bank of Zimbabwe, development partners, donors, bilateral agencies, and NGOs. Recognising the situation's urgency, UNICEF is developing a comprehensive cholera response plan. The estimated funding requirement for Zimbabwe stands at US$ 8,047,500 to provide essential Water, Sanitation, and Hygiene (WASH) services, healthcare, risk communication, nutrition, child protection, and education services to women and children [9]. Nevertheless, a substantial funding gap remains, underscoring the financial challenges of managing the outbreak.

### Migration and Labor Impact

The cholera outbreak has incited a significant migration of people from affected areas, resulting in a loss of labour and economic input and a decline in financial income. Moreover, these migrations risk introducing cholera to other areas or communities previously untouched by the outbreak. For those who run food carts, the fear of selling contaminated food looms large as flies swarm their cooking areas [10]. Meanwhile, some patients have bravely shared their personal experiences, shedding light on the profound toll and challenges faced by those afflicted by the outbreak [2].

## Current Efforts Towards Mitigation

In response to the 2023 cholera outbreak in Zimbabwe, the government and non-governmental organisations (NGOs) have launched extensive efforts to combat the disease. These combined efforts encompass a range of strategies to mitigate the outbreak's impact and prevent its spread.

### Government-Led Initiatives

The Zimbabwean government's response has been multifaceted and swift. They established a Cholera Treatment Center in Chegutu town, a critical step in providing immediate medical care to those affected [10]. To curtail the spread of cholera, the government imposed restrictions on large gatherings, implemented heightened surveillance at ports of entry, and enforced specific measures to reduce the risk of transmission. Funeral attendance has been limited to no more than 50 people, and physical contact, including handshakes, has been discouraged. Furthermore, participation in outdoor church camps and open markets has been discouraged to minimise the risk of exposure [2]. An inter-ministerial committee, coordinated through the Cholera Secretariat of Zimbabwe, convened a National Task Force for Cholera Elimination. This task force developed a comprehensive Multi-Sectoral Cholera Elimination Plan, guided by the Global Task Force on Cholera Control's (GTFCC) global roadmap. Additionally, the government has worked on strengthening surveillance and laboratory capacity to enable early detection and rapid responses to cholera outbreaks [11]. However, it must be noted that while these steps have helped, there are limitations to their effectiveness. For instance, establishing a Cholera Treatment Center has been a critical response to cholera outbreaks. However, its effectiveness has been compromised by various factors [2]. First, the centre's capacity is often overwhelmed during the surge in cholera cases during a large-scale outbreak. This limitation becomes more pronounced when a sudden and significant patient increase requires treatment. As a result, overcrowding, longer waiting times, and the inability to provide timely patient care occur, leading to increased morbidity and mortality rates. Similarly, while immediate responses to cholera outbreaks are crucial, it is essential to acknowledge that cholera control is an ongoing process. Sustainable efforts are required to address cholera's root causes and prevent future outbreaks. This involves investing in long-term solutions such as improving access to clean water and sanitation infrastructure, which is critical for cholera prevention. However, sustainability efforts have faced challenges regarding financial resources, political commitment, and coordination among various stakeholders. Addressing these limitations is crucial to achieving more effective and comprehensive cholera mitigation strategies in the country.

### WHO Support

The World Health Organization (WHO) has played a vital role in supporting Zimbabwe’s efforts to combat cholera. During their visit in February 2023, WHO provided essential aid, including cholera kits containing medicines and laboratory reagents. They also offered technical support to enhance the capabilities of frontline health workers, helping to improve their capacity to respond effectively to the outbreak. WHO highlighted the key challenges that required immediate attention, emphasising the importance of collaborative efforts among WHO, Zimbabwe, and other partners to address the cholera outbreak [12] effectively. However, it is important to acknowledge that while WHO’s aid is crucial in the short term, heavy reliance on external aid can pose a challenge to sustainability. Effective responses to cholera outbreaks require short-term training and a focus on continuous professional development, infrastructure improvement, and resource allocation. For long-term effectiveness, Zimbabwe must develop self-reliance and build its capacity to manage cholera outbreaks independently. In addition, the sustainability of these improvements is limited if there is insufficient investment in building local capacity.

### IFRC and UNICEF Contributions

The International Federation of Red Cross and Red Crescent Societies (IFRC) has supported Zimbabwe through various initiatives. They led training events covering Oral Rehydration Therapy and assisted in organising Oral Cholera Vaccine Campaigns. In collaboration with the Zimbabwe Red Cross Society (ZRCS), IFRC provided resources such as tents and volunteers trained in shelter construction for Cholera Treatment Centers. They also distributed water treatment products to communities and conducted awareness campaigns to raise cholera awareness. The United Nations, including UNICEF, were crucial in mobilising resources for the cholera response. To address the financial aspect, the government set aside $24 million to support these efforts [13, 14].

These comprehensive efforts, led by government and NGO initiatives, form a robust response to the cholera outbreak in Zimbabwe, encompassing medical treatment, surveillance, public health measures, infrastructure improvement, and community empowerment. The collaboration among various stakeholders, including international organisations, highlights the dedication to effectively combat the disease and protect the health of Zimbabwe's population. These efforts aim to mitigate the outbreak's impact and prevent its spread. While the collaborative efforts involving government and international organisations are commendable, challenges related to dependency on foreign aid, resource sustainability, local capacity building, community engagement, coordination, financial allocation, and long-term prevention efforts need to be addressed for a more effective and sustainable response to cholera outbreaks in Zimbabwe. Balancing short-term emergency response with long-term public health infrastructure improvements is crucial for comprehensive cholera control in Zimbabwe.

## Policy Recommendations and Future Directions

In the ongoing battle against cholera in Zimbabwe, it is imperative to effectively outline policy recommendations and future directions to bolster prevention and control measures. Key aspects of these recommendations revolve around gleaning insights from past cholera outbreaks and adopting globally proven strategies.

### Learning and Adaptation

In the ongoing battle against cholera in Zimbabwe, it is imperative to effectively outline policy recommendations and future directions to bolster prevention and control measures. Key aspects of these recommendations revolve around gleaning insights from past cholera outbreaks and adopting globally proven strategies. Learning from previous experiences is crucial, emphasising the importance of implementing strategies that have yielded success on a global scale. For example, analysing past outbreaks might reveal that specific regions or communities consistently experience higher cholera incidence due to inadequate sanitation infrastructure. Moreover, it instils a culture of continuous improvement and adaptation in the country's response to cholera. Emphasising that no outbreak should be viewed as a failure but as an opportunity for growth is key. Post-outbreak reports can be institutionalised as a standard practice, ensuring that lessons learned are integrated into future cholera prevention and control strategies.

### Communication and Engagement

Effective communication and engagement play pivotal roles in the battle against cholera. The policies emphasise the importance of risk communication, a fundamental element. Enhancing risk communication stands as a fundamental element. Public awareness campaigns should be comprehensive, reaching all population segments through diverse communication channels [15, 16]. Public awareness campaigns can be done through locally accepted media channels, such as radio and community meetings, to reach different population segments. These campaigns can emphasise handwashing with soap and safe food handling. The primary goal is behavioural change, instilling knowledge about proper hygiene practices, safe water handling, and food preparation to minimise contamination risks. In high-risk communities, residents could establish local health committees responsible for monitoring water quality and hygiene practices. This empowers the community to take ownership of cholera prevention. Moreover, stakeholder engagement is another critical component. Effective cholera prevention and control require collaboration between numerous entities, including the National Society, the Ministry of Health, international organisations, NGOs, and local community groups. These partnerships facilitate resource mobilisation, enabling stakeholders to combine their strengths, share expertise, and coordinate a comprehensive response.

### Standardisation and Evaluation

Standardizing cholera case definitions is fundamental to ensure data accuracy. These definitions create a common language for healthcare providers and epidemiologists, facilitating the accurate diagnosis and reporting of cholera cases. However, it is equally crucial for these definitions to adapt to local contexts, as cholera's presentation can vary regionally. Regularly evaluating interventions is vital for data-driven decision-making and guiding adjustments. These assessments comprehensively analyse various sources' data to gauge intervention effectiveness [17]. Furthermore, adaptable interventions are imperative to address specific challenges, and local communities actively participate in the adaptation process. Cholera control strategies should not remain static. Continuous evaluation is essential to assess the effectiveness of implemented measures. Regular assessments and data analysis are critical to identifying gaps and areas for improvement. Regular surveillance system reviews can help identify emerging trends, enabling a proactive response to potential outbreaks.

### Resource Allocation and Capacity-Building

Efficient resource allocation is vital, ensuring that necessary funding, medical supplies, and logistical support are readily available to respond to outbreaks. Rapid resource mobilisation can make a significant difference in controlling cholera's spread. Creating a dedicated fund for cholera preparedness and response can ensure that financial resources are readily accessible when needed. Capacity-building efforts for healthcare workers are instrumental in creating a coordinated, data-driven response to cholera outbreaks. Training and skill development must be implemented to empower healthcare professionals to deliver quality care and conduct effective surveillance. For instance, training in advanced epidemiological techniques enables epidemiologists to detect patterns in the data and implement timely interventions. Ongoing training programs can enhance healthcare workers' knowledge and skills in cholera case management, improving patient outcomes. Regular drills and simulations for the response team are essential to ensure that teams are familiar with response protocols and can work cohesively under high-pressure situations. These efforts foster a more robust and adaptive system for managing cholera effectively.

## Conclusion

The 2023 cholera outbreak in Zimbabwe serves as a reminder of the ongoing vulnerability of communities facing challenges related to clean water, sanitation facilities, and public health infrastructure. While the immediate health impact has been devastating, the repercussions extend beyond these numbers. The outbreak has strained an already fragile healthcare system, exacerbating issues related to endemic diseases, malnutrition, and limited access to healthcare, particularly for vulnerable populations. As the world continues to battle preventable waterborne diseases like cholera, it is imperative to draw lessons from the Zimbabwean experience. Key policy recommendations and future directions encompass risk communication, stakeholder engagement, standardisation, evaluation, resource allocation, and capacity-building. These recommendations provide a roadmap for bolstering prevention and control measures, emphasising the importance of adaptive, data-driven responses. The collaboration among various stakeholders reflects a dedicated commitment to protect public health and mitigate the outbreak's impact.

## Data Availability

Data sharing is not applicable to this article as no datasets were generated or analysed during the current study.

## Abbreviations

MoHCC: Ministry of Health and Child Care
WHO: World Health Organization
IFRC: International Federation of Red Cross and Red Crescent Societies
NGOs: Non-Governmental Organizations
ZRCS: Zimbabwe Red Cross Society
WASH: Water, Sanitation, and Hygiene
GTFCC: Global Task Force on Cholera Control
COVID-19: Coronavirus Disease 2019
UNICEF: United Nations International Children's Emergency Fund
